# Pennsylvania State Veterinary Medical Association

**Published:** 1900-11

**Authors:** 


### SOCIETY PROCEEDINGS.

### PENNSYLVANIA STATE VETERINARY MEDICAL ASSOCIATION.

THE semi-annual meeting was held at Erie, September 18, 1900. It was opened by President Harger at 10.30 A.M. At roll-call the following members were noted as present: Drs. J. Buckham, Beaver Falls; M. J. Chrisman, Sugar Grove; H. Emery, Pittsburg; S. J. J. Harger, Philadelphia; F. F. Hoffman, Brookville; W. Horace Hoskins, Philadelphia; J. B. Irons, Erie; George B. Jobson, Franklin; C. J. Marshall, Philadelphia; C. C. McLean, Meadville; W. A. Meredith, Corry; Leonard Pearson, Philadelphia; E. C. Porter, New Castle; E. M. Ranck, Philadelphia; A. W. Radley, Bethlehem; A. W. Weir, Greenville.

Among the visitors were: Drs. T. M. Brackett, Erie; J. Bryce, Erie; Mr. Churchill, Erie; Drs. A. E. Cunningham, Cleveland, Ohio; Harry E. Dunn, Erie; Mr. J. R. Ferguson; Drs. John Gobin, North Collins, N. Y.; C. Z. Laberge, Beaver Falls; R. M. Phelan, Sharon; Hunter Smith, Rowesville; Wm. M. Wilson, Hartstown.

Reading of minutes of the annual meeting was dispensed with on account of the shortness of time and length of the minutes.

Next in order of business was the President's address, which was well presented by Dr. Harger.

### *Address of the President.*

This morning we open the semi-annual meeting of the Pennsylvania State Veterinary Medical Association. While I speak this name allow me to suggest that it would be more in accordance with good grammar and with the true scope of the work of our profession if the by-laws were so amended as to omit the word "medical."

Not having had an opportunity before to extend my obligations to you for electing me to this position, I shall now take occasion to do so and endeavor to promote the work of this Association with the same zeal that my predecessors have displayed.

The last annual meeting in Philadelphia, one of the most, if not the most, successful in our history, is still fresh in our minds. The semi-annual meeting is by many, though erroneously, considered of second importance, and consequently the attendance is usually rather small. I regret that at this meeting, as well as at the semi-annual gatherings in the past, so many members who could ably afford to take this journey make themselves conspicuous by their absence. Many of the members from the western part of the State go to the annual meeting in Philadelphia, and the members from the latter section of the State can be expected at least once a year to leave their own bailiwick and pay their respects to their colleagues as well as to the organization as a body at some distant point. If there is not sufficient apparent inducement for them to participate they should, by their presence and collaboration, create such inducement.

Far from being a pleasure visit to a distant part of the State, the semi-annual as well as the annual meetings have for their prime object the enhancing of the work and the principles which we represent. Numerous invitations have been sent to veterinarians not members. Almost every veterinarian in this section of the State has received an invitation from us, thinking that on account of the nearness of the place of meeting they would take advantage of this opportunity and give the more active members a chance to arouse some enthusiasm in Association work and convince them of the power and influence which accrue from the collaboration of many individuals combined into one harmonious whole.

I find, after consulting the veterinary registry of this State as well as from many personal acquaintances that I have made, that the veterinarians in the counties of this section are quite numerous; that professionally they are well advanced; they take an interest in their profession; they are active participants in our Association; they strive to drown worthless, empirical ideas; they work harmoniously with the powers that be in the control of animal diseases and the exploitation of the live-stock interests.

We have accomplished much, but we must not be egotistical. This we can see by comparing our position of the present with that of twenty-five years ago. Our profession has sprouted and grown rapidly; but, comparatively speaking, it has still not yet reached its full manhood; in its skeleton we still see traces of the primitive centres of ossification, evidence of incomplete development. In order to effect a complete coalescence of these into one solid frame around which the nerves and sinews of our work are grouped, enabling these to produce their maximum results, it requires every effort of which the phenomena of assimilation and growth are capable. The identity of many diseases remains to be determined—many of those which are now grouped under the same nomenclature are to be differentiated; veterinary science must play an important factor in hygiene—in the preservation and amelioration of the public health; laws must be enacted and amended for controlling the spread and eradication of animal diseases within this State and in interstate traffic; the slaughter of animals, and meat and milk-inspection require the supervision of the expert veterinarian; the transmission, through the consumption of animal products, of diseases from the lower animals to man must be guarded against, and the unnecessary sacrifice of human lives through these agencies must be prevented. The time will come when boards of health must associate with themselves a veterinarian whose province it is to trace sporadic deaths as well as epidemics to the cow-stable, the creamery, or the slaughter-house. We have a State Veterinary Examining Board that requires your assistance to remedy its weak points; also a Live-stock Sanitary Board, well known to you, which requires your coöperation. On legislative matters you will be enlightened during these proceedings. The veterinarian should be a guiding factor in the breeding of animals and their selection, encouraging the elimination of those unfit to have offspring; also in the management of animals in competition for prizes, as in horseshows and fairs where such exist. The veterinarian can profitably associate himself with medical societies and point out to the physician facts and conditions in comparative pathology of which the average medical practitioner may not take recognition. In these and in many other varied ways the veterinarian may make himself a potent factor in the interests of his community, his profession, and in advancing his own position.

The safety of our future has, by some alarmists, been questioned. Shall we die or shall we live? Our anticipations of the future need not be so gloomy; we are not so near the threshold of annihilation. From what you may have heard you may imagine that the horseless carriage has sealed our doom; that private as well as public stables and horseshoeing shops have all closed up, and that the streets of the large cities are lined with horseless wagons. But if one looks for these machines he will be disappointed; he will see a few here and there, but not in sufficient numbers to cause him to look for another vocation. The end is not yet. The practitioner is at present as prosperous and has as much work to do as he had at any time in the past. The value of horses has increased sufficiently to induce owners to seek the services of a doctor in questions of soundness as well as in sickness. Breeding operations are most active. The demand is greater than the supply. Foreign governments, such as England and France, have for several years been draining this country of cavalry and artillery horses. Now, Germany comes to the aid of the breeder and the dealer by placing an order for 30,000 horses for the German army. True, we have at the present time more veterinarians than existed twenty years ago. There is more competition. We must be more active and accurate in our work. But competition makes better men of us; it is one of the essentials to progress. There is still light ahead of us. As old fields of labor are closed new ones will open themselves before us. The vicissitudes of time, the wave-like line now indicating prosperity, then poverty, will eventually seek a normal level on which we may stand with safety.

With these few remarks at this time I shall remain contented. At our annual meeting I expect to have the opportunity to address you with remarks containing more details and covering a larger field.

The following members were elected and introduced: W. P. Bagnall, D.V.S. (Chicago), Warren, Pa.; J. Bryce, V.S. (Ontario), Erie, Pa.; W. H. Fry, V.S., Pine Grove Mills, Pa.; C. Z. Laberge, M.D., Beaver Falls, Pa.; R. M. Phelan, V.S. (Ontario), Sharon, Pa.; J. R. Phillips, V.S. (Ontario), Northeast, Pa.; W. M. Wilson, V.S. (Ontario), Hartstown, Pa.

Next in order was Secretaries' reports. Dr. Ranck read a communication from Dr. W. H. Wilson, of Hatboro, Pa., to Dr. W. H. Ridge, in reference to being reinstated in the Association. Referred to the Executive Committee.

*Reports of Committees.*

*Legislation.* W. L. Rhoads offered a communication: Being a member of the Legislative Committee, and having no report to make of work done by our committee, that I know of, that is of importance to the profession or of interest to those assembled at Erie, I would suggest that the Association take up at this time the subject of registration and inspection of stallions standing for service within the State. The day is at hand when the farmer and horseowner will see the necessity of breeding, and we as a profession owe it to ourselves and the community to shield the unwary and careless from the error of breeding to inferior males, and I therefore offer the following resolution:

*Resolved*, That the President at the Erie meeting appoint a committee of three or five to take up this subject and formulate a memorial to be presented at our March meeting, there to be finally acted upon.

As the New Jersey Association is in the field, endeavoring to have the

1901 meeting of the American Veterinary Medical Association meet at Atlantic City, I would also offer the following:

*Resolved*, That the Pennsylvania State Veterinary Medical Association do all in its power as an Association and the members individually to assist the New Jersey veterinarians in persuading the Executive Committee of the American Veterinary Medical Association to hold the annual meeting of said Association at Atlantic City in 1901.

In the discussion of this report Dr. Hoskins spoke of the advantages of Atlantic City for the meeting of the American Veterinary Medical Association in 1901.

Dr. Irons spoke about a previous law being in vogue in reference to registration of stallions, having been brought about through the efforts of the Tri-State Association, and was enacted as a State statute in 1893.

Motion by Dr. Hoskins, that we recommend Atlantic City for the Amerian Veterinary Medical Association meeting in 1901; carried.

*Intelligence and Education.* An excellent report was presented by the Chairman, Dr. Felton, who was absent, and his report was read by the Secretary:

The middle of the year finds the veterinary profession in Pennsyivania in a very prosperous condition. The advance in the price of horses and the advent of good times generally have rendered horseowners sufficiently solicitous for the welfare of their animals to employ skilled treatment for their ailments, instead of depending upon the stable-boss or the friend who volunteers his services. Dealers declare that high-class horses are once more in great demand, and that the price is advancing rapidly. Judging from the great activity in the horse-markets of Philadelphia, the "horseless age" is farther off than ever, and we can say again "the horse is king; long live the king!" Recently, a prominent dealer of Philadelphia sailed for England with twenty-eight head of fine coach-horses, all ordered in advance, for which he expects to get from $400 to $700 each. The best type of heavy draught-horses, known as brewers' horses, are bringing $500 a pair, twice what they did two years ago, and are very scarce at that figure. Big horses are scarce in the Western markets on account of the heavy shipments that have been made to England, Germany, and South Africa. Pennsylvania dealers and breeders are much encouraged over the outlook for the next few years. Their horses always command a better figure than the Western horses, as they are considered to be acclimated and less subject to disease.

The farmers of Pennsylvania are becoming every year more interested in dairy farming as a source of revenue, and it has been noted that at the farmers' expositions and granges held throughout the State the dairy interests have been a prominent theme of discussion. The question of the illegal sale of oleomargarine has become a burning theme of discussion, it being alleged by the representatives of the "Pure Butter Protective Association" that 12,000,000 pounds of oleomargarine are sold annually in the State of Pennsylvania, not as oleomargarine, but colored in defiance of the law of May, 1899, and sold as pure butter in competition with the genuine article. It is further alleged that such sale entails a loss to the farmers of $4,000,000 annually. If this be true, it is no wonder that the farmers have risen up in their wrath and demanded that the law shall be enforced. Grave charges have been brought against former Dairy and Food Commissioner Levi Wells, who resigned under the pressure brought to bear upon him. His successor, Jesse Cope, and the Secretary of Agriculture, Prof. John Hamilton, have also come in for a measure of condemnation from the farmers, being charged with laxity in the prosecution of violators of the oleo law and subservience to a political machine which levies tribute from the oleo trust. In the mass of crimination and recrimination with which the papers are filled at the present time it is difficult to sift out the truth; but as Secretary Hamilton and Commissioner Cope have announced their determination to honestly enforce the law, it would be well to give them a fair chance. The conflict now going on between the Department of Agriculture and the dairy interests is not an edifying spectacle, and raises the question whether in making the department a political organization we have not taken a step backward. The old State Board of Agriculture was non-partisan, being composed of representatives from both political parties.

We are pleased to note that the proper care of domestic animals, in regard to hygiene, shoeing, etc., and the subject of good roads, have been fruitful themes of discussion at the annual grange meetings. In connection with the dairy interests we note the following communication from Consul Nelson, who writes from Bergen, May 30, 1900, as follows: "Cheese from pasteurized milk has until lately been considered almost impossible to produce, and dairymen have been at a loss how to use the churn milk, which has been sold as feed for pigs or thrown away. A short time ago a chemist at Stockholm, Dr. Frans Elarder, succeeded in effecting a preparation which solved the above-mentioned difficulties. Owing to this discovery, which he has named 'caseol,' palatable and nourishing cheese, free from the bacillus tuberculosis, can now be made from pasteurized skim-milk. This preparation has, moreover, the excellent quality of rendering cheese more digestible. Several dairies in London have made experiments with 'caseol' with the same favorable results. I will gladly procure samples of 'caseol' for any of our dairymen who desire to make trial of it." At the Farmers' National Congress, held at Colorado Springs, August 22, 1900, Mr. George Whitaker made an address on "Dairying," in which he said: "The annual value of the dairy products of the nation is in round figures $500,000,000. The production of butter has increased more rapidly than the increase in population. The census of 1850 reported 13.51 pounds per capita; the census of 1890, 19.24 pounds. One-half of the butter production is in seven States. The largest butter State is Iowa, making 10.4 per cent. of the country's production; next comes New York with 9.3 per cent, and Pennsylvania third with 8 per cent. Exports during the last ten years have fluctuated between 6,000,000 and 31,000,000 pounds. For the year ending June 30, 1899, the amount was 20,000,000 pounds. The production of milk for consumption is the second largest branch of the industry, using the product of 5,500,000 cows. In Boston, with a population of three-fourths of a million people, the large wholesalers brought into the city in the year 1899 about 95,000,000 quarts. Estimating the amount from other sources at 25,000,000 quarts, we have a total of 120,000,000 quarts. The farms netted on an average at least two and a half cents per quart, which makes the business worth to them $3,000,000. In New York, with twice the population of Boston, figures based upon the amount required to furnish the sale of milk, cream, and condensed milk used in the city are placed at

584,000,000 quarts per year, which, at two and a half cents per quart, amounts to $14,000,000." According to Secretary Hamilton, 200,000,000 pounds of butter are consumed annually in Pennsylvania, of which 100,000,000 are produced by the farmers. Mr. Eastburn Reeder, however, claims that these figures are too large, as they would mean a consumption of one pound of butter a week for every man, woman, and child in the State, which is excessive. According to the census of 1890, 76,336,012 pounds of butter were produced in the State, which was 3,000,000 less than was produced in 1880. The figures from the recent census are not yet obtainable, so we are not able to verify the above statements.

Recently a new method of building up the nutrition of tuberculous patients by feeding them on the strippings of cow's milk fresh from the cow has been adopted by a number of prominent lung specialists. Wonderful results are claimed for this treatment, and as it requires that the cow shall be perfectly free from disease, especially tuberculosis, it is a matter of great interest to the veterinarian. The war against tuberculosis is being carried on unrelentingly. More rational methods of treatment are being advocated. Disinfection and the removal of unsanitary surroundings are being more strenuously urged. The building of sanitariums in unexceptionally healthful locations under State supervision, where patients can be treated under the best possible conditions, is a long step in advance. It is gratifying to the veterinarian to know that more and more the medical profession is looking upon him as an ally in this fight, and that in guarding the purity of the meat-supply and milk-supply his services are indispensable.

The Bureau of Animal Industry has done an extremely valuable work in preparing and distributing free of charge the vaccine for blackleg. This malady in some States of the Union destroys more cattle than all the other causes combined. Texas, Kansas, Nebraska, Colorado, and the Dakotas suffer very severely. In these States there had formerly been a loss of from 10 to 20 per cent. of yearling stock annually. Since the vaccine has been used the losses have been reduced to less than one-half of 1 per cent. Four years ago 50,000 doses were sent out by way of experiment, and the results obtained were so remarkable that 350,000 doses were distributed in the following year in response to requests. In the third year 500,000 doses were given away, and in 1900 over 1,000,000 doses will be sent out. We are glad to learn that the Live-stock Sanitary Board will have $40,000 to expend this year in the work which they are so efficiently carrying out under the able direction of Dr. Leonard Pearson.

In the July number of the JOURNAL OF COMPARATIVE MEDICINE AND VETERINARY ARCHIVES we find a communication from Dean Conkey, of the Grand Rapids Veterinary College, in which, in our estimation, he lowers himself beneath the level that a man in his position should occupy. We are glad that it is not necessary for the Dean of a Pennsylvania college to lay a wager as to whether he possesses a certain amount of knowledge, and we believe he would be courteous enough, even in the heat of controversy, to give a man his proper title. Drs. M. P. Ravenel's and D. J. McCarthy's article in the July JOURNAL, entitled the "Rapid Diagnosis of Rabies," is an extremely valuable contribution to our English literature on this subject, and we trust it will stand the test of further experiment and give to the veterinarian a new and sure method of diagnosing this disease in doubtful cases. Dr. L. O. Lusson, of Ardmore, Pa., reports three outbreaks of rabies from February to July of this year. Dr. Lusson reports to me the following case, which, if his diagnosis be correct, will modify somewhat our views of this disease: Fox terrier, aged five months, bitten in the nose by a rabid pug; seven days after the following symptoms were noticed: Two hours of intense excitement, immediately followed by paralysis of the throat, the puppy being unable to eat or drink; hyperæsthesia of one forepaw and partial paralysis of the hindquarters. Hyperæsthesia lasted about two days and paralysis of the throat about one week, as did staggering gait. Treatment: During the period of excitement used a very hot bath; confined patient in a dark box, with plenty of fresh water and soft food. At the end of seven days paralysis of the throat gradually subsided and the patient began taking water and food. Not at any time was he aggressive. Diagnosis, mute rabies. Dr. Lusson adds: "I am fully convinced that many of these cases in young and vigorous dogs will recover." We have seen many cases of mute rabies, and all that were allowed to live died about the fourth or fifth day of general paralysis and spasm of the muscles of respiration. It is true that we did not try the hot bath, which is according to the treatment of Dr. Baisson, for which so much is claimed in human practice. It will be very interesting to follow up the history of this case and see if the cure is a complete one, or whether the animal will be subject to relapses. Further experiments ought to be made along this line. The article by Drs. Leonard Pearson and M. P. Ravenel in the August JOURNAL on "A Case of Pneumonomycosis due to Aspergillus Fumigatus," is also a very valuable contribution to our literature, and the veterinary profession is under great obligation to them for the painstaking care and deep research which were necessary in order to make the diagnosis of this hitherto unsuspected disease among cattle in this country. Drs. Salmon and Huidekoper have been untiring in their efforts to further the cause of the veterinarian in the army. Their presentation of the case before the Committee on Military Affairs of the House of Representatives was a masterly one, and it is sincerely hoped that their labor will not be in vain, but will be fruitful of good results.

(To be continued.)

