# Correction

**Published:** 1901-01

**Authors:** 


### CORRECTION.

Volume XXI., No. 11, page 707, line 24, Dr. Frans Elarder should read Dr. Frans Elander; page 709, line 19, Dr. Baisson should read Dr. Buisson.

